# Random forest classifier improving phenylketonuria screening performance in two Chinese populations

**DOI:** 10.3389/fmolb.2022.986556

**Published:** 2022-10-11

**Authors:** Yingnan Song, Zhe Yin, Chuan Zhang, Shengju Hao, Haibo Li, Shifan Wang, Xiangchun Yang, Qiong Li, Danyan Zhuang, Xinyuan Zhang, Zongfu Cao, Xu Ma

**Affiliations:** ^1^ National Human Genetic Resources Center, National Research Institute for Family Planning, Beijing, China; ^2^ Graduate School of Peking Union Medical College, Beijing, China; ^3^ Gansu Province Medical Genetics Center, Gansu Provincial Clinical Research Center for Birth Defects and Rare Diseases, Gansu Provincial Maternity and Child-Care Hospital, Lanzhou, China; ^4^ The Central Laboratory of Birth Defects Prevention and Control, Ningbo Women and Children’s Hospital, Ningbo, China

**Keywords:** newborn screening, MRM, machine learning, phenylketonuria, random forest classifier

## Abstract

Phenylketonuria (PKU) is a genetic disorder with amino acid metabolic defect, which does great harms to the development of newborns and children. Early diagnosis and treatment can effectively prevent the disease progression. Here we developed a PKU screening model using random forest classifier (RFC) to improve PKU screening performance with excellent sensitivity, false positive rate (FPR) and positive predictive value (PPV) in all the validation dataset and two testing Chinese populations. RFC represented outstanding advantages comparing several different classification models based on machine learning and the traditional logistic regression model. RFC is promising to be applied to neonatal PKU screening.

## Introduction

Phenylketonuria (PKU [MIM: 261600]) is an autosomal recessive genetic disease, which is one of the common disorders of amino acid metabolism (Yan et al., 2019). It is also one of the diseases for newborn screening (NBS) in China. The incidence of PKU in China is 1/10,701, with a higher incidence in the north than in the south (Wang et al., 2015). The incidence of PKU in Hainan province of China is approximately 1/81,967 (Huang et al., 2021) but 1/3,420 in Gansu province (Wang et al., 2015). Due to the high cost of gene detection, some methods for PKU screening were used such as the Guthrie test (Guthrie and Susi, 1963) and high performance liquid chromatography (HPLC) (Moretti et al., 1990) in the early days after birth. Tandem mass spectrometry (MS/MS) is currently used in many countries to screen inborn errors of metabolism (American College of Medical Genetics Newborn Screening Expert Group, 2006; Lindner et al., 2011). In most countries around the world, PKU screening is performed by evaluating phenylalanine (PHE) and tyrosine (TYR) levels in neonatal dry blood spots (DBSs) by LC-MS/MS (Blau et al., 2014). In clinical, newborns with PHE concentration more than 120 μ mol/L will be recalled, and then genetic testing will be carried out to confirm. This screening method brings a high false positive rate, which can waste a lot of medical resources and even bring panic to the involved families. Therefore, there is great clinical value to improve the accuracy for PKU screening.

Machine learning is the science of artificial intelligence and has been widely used in medicine (Deo, 2015). For example, there are many important applications in the establishment of cancer mutation spectrum, cancer research and nursing care, and the diagnosis and prognosis of cardiovascular and cerebrovascular diseases (Muiños et al., 2021; Meropol et al., 2021; Savarraj et al., 2021). It also plays an important role in the screening of neonatal genetic metabolic diseases (Baumgartner et al., 2004). For example, a random forest machine learning classifier was used to establish NBS models for glutaric acidemia type 1 (GA-1), methylmalonic acidemia (MMA), ornithine transcarboxylase deficiency (OTCD) and very long-chain acyl-CoA dehydrogenase deficiency (VLCADD) (Peng et al., 2020). Further, several studies in PKU screening have attracted more attention. A logistic regression model was constructed for PKU screening, in which sensitivity reached 95%–100% and PPV increased from 19.14% to 32.16% (Zhu et al., 2020). In addition, feature selection strategy was used to obtain the optimal biomarkers and reduce the false positive proportion of PKU (Chen et al., 2013).

However, PKU screening based on the model constructed by machine learning methods has not been widely used in practice. Most hospitals still follow traditional methods for PKU screening. As a result, it is particularly urgent to develop and fine-tune classification models for rare but treatable metabolic diseases such as PKU. It aims at both reducing false positive cases and eliminating false negatives, in order to detect the infants and children with PKU quickly and accurately. In this study, we applied RFC method to improve PKU screening performance with excellent sensitivity, FPR and PPV in two Chinese large populations.

## Materials and methods

### Metabolic data

The population level newborn screening data of small molecule metabolites were from Gansu Provincial Maternity and Child-care Hospital (GPMCH) in the northwestern China and Ningbo Women and Children’s Hospital (NWCH) in the southeastern China. Small molecule metabolites including 10 amino acids and 31 acylcarnitines of each newborn were obtained from blood by MS/MS. All newborns consist of 43 features, including 41 small molecule metabolites and two ratios which are the traditional biomarkers PHE/TYR and the new potential biomarker MET/PHE [16]. Newborn samples will be divided into two categories, that PKU patients and normal samples without PKU (Non-PKU). All PKU newborns and children have a clear causative pathogenic variant verified by Sanger sequencing or Next-generation sequencing. To protect personal privacy, personal information of all samples was deleted.

### Data processing and description

All the samples with other metabolic disorders were excluded for all the datasets to avoid misleading the prediction results. Then, all features were normalized with a multiple of the median (MOM) to avoid systematic errors. The median of every feature is first calculated. Then, the original value is divided by the median to obtain the normalized value, which called MOM value (Yang et al., 2021).

**FIGURE 1:**
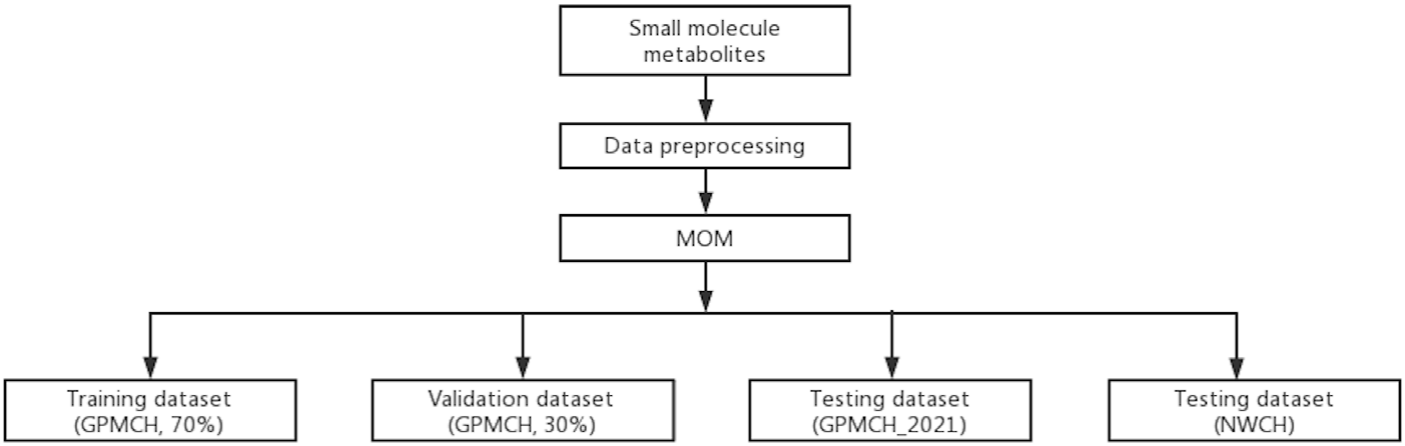
The flow chart of dataset processing and distribution.

During data preprocessing, 163 PKU patients with treatment information and 565 samples with other metabolic disorders were excluded. The total datasets described in model were all preprocessed. In GPMCH population, 22,867 records from 2015 to 2020 were randomly split into the training and validation datasets at a 7/3 ratio after processing. Consequently, the training dataset contains 132 PKU patients and 15,874 Non-PKU samples for fitting the model, the validation dataset contains 69 PKU patients and 6,792 Non-PKU samples for optimizing the model. Two testing datasets were used to evaluate the performance of the model. One testing dataset (GPMCH_2021) included 9 PKU patients and 1,398 Non-PKU samples from January to May 2021. The other testing dataset (NWCH) included 16 PKU patients and 392,177 Non-PKU samples from 2014 to 2020. The processing steps of these datasets are shown in Figure 1 and descriptive statistics of 43 biomarkers used in the research are depicted in Supplementary Table S1.

### Machine learning models

PKU screening models were built using six machine learning methods, including Multilayer Perceptron (MLP), Decision Tree (DT), Stochastic Gradient Descent (SGD), Logistic Regression (LR), K-Nearest Neighbor (KNN) and RFC. All models were built with Scikit-learn-0.23.2 in python and optimized by adjusting parameters.

Logistic regression analysis 3 (LRA3) is a classification model developed by Zhixing zhu et al. with good sensitivity, specificity and PPV for PKU screening [16]. The formula of this model is as follows:

Logit of model z=0.7722–13.2300·Met/Phe+0.0010·Phe–0.0090·Tyr (1)

### Random forest classifier

RFC is a highly flexible supervised classification tool. The classification model trains and predicts samples with multiple decision trees (Breiman, 2001). It can avoid the phenomenon that a single decision tree is prone to over-fitting and improve prediction accuracy. The process of RFC is summarized as follows:

1) Among the n samples of the original training dataset, i samples are randomly sampled with replacement. All training samples of each classification tree form a new training dataset.
2) For each training dataset, a classification and regression tree algorithm is used to construct the classification tree without pruning leaves is generated separately. At each internal node of the tree, m features (m≤M) are randomly selected from m features as the candidate attributes of the splitting node, and the optimal splitting genus is selected from M candidate attributes to split the node. This classification tree is fully grown to generate the largest tree, so that the impurity of each leaf node is minimized and pruning operation is not carried out.
3) There are n classification trees in the RFC model and each tree has a category determination result, the category with the most votes is designated as the final output.

The RFC model was built by fine-tuning its parameters in the training dataset, including the number of trees in the forest, the maximum depth of the tree, the minimum number of samples required to split the internal nodes, the minimum number of samples required for the leaf nodes and measuring the performance of the trained model in the validation dataset. Due to the imbalance of the data, we set category weights with low weights for large sample sizes and high weights for small sample sizes. To obtain the optimal model, “Grid Search” of Python library is used to fine-tune parameters. The ideal requirement in clinical is to detect all PKU patients with excellent PPV at the same time. When the new sample enters the RFC model, each decision tree of RFC gives its own disease status of PKU. By integrating the disease status of each decision tree and adopting a simple voting method of minority obeying the majority, the RFC model determine whether the sample has PKU.

### Feature importance

Gini impurity is used to rank the relative importance of each feature. It is the probability of misclassification of randomly selected elements after randomly marking according to the class distribution in the dataset. In RFC, feature importance represents the sum of Gini impurity reduction of all nodes split on features. The smaller the Gini impurity, the smaller the probability that the selected samples in the dataset are misclassified, and the better the feature.

### Performance evaluation

This study is a binary classification problem with random forest. The confusion matrix is used to view the correct and wrong recognition of each kind of samples (Table 1).

**TABLE 1:** Confusion matrix.

Confusion matrix		True value	
		PKU	Non-PKU
Predict value	PKU	True positives ( TP )	False positives ( FP )
	Non-PKU	False negatives ( FN )	True negatives ( TN )

Pearson chi-square test is a hypothesis testing method based on the chi-square distribution, inferring whether two categorical variables are correlated or independent of each other according to the sample data. In this study, it is applied to test the independence of true value and predict value in the confusion matrix.

Then the performance evaluation indices calculated from the confusion matrix are as follows:

$$\begin{array}{c} Accuracy=\frac{TP+TN}{TP+FP+TN+FN}\\ Sensitivity=Recall=\frac{TP}{TP+FN}\\ \begin{array}{c} Specificity=\frac{TN}{FP+TN}\\ \begin{array}{c} PPV=Precision=\frac{TP}{TP+FP}\\ FPR=\frac{FP}{FP+TN} \end{array} \end{array} \end{array}$$ (2)

We also plotted precision recall (PR) curve and receiver operating characteristic (ROC) curve to evaluate our model, meanwhile calculated the average precision (AP) and the area under curve (AUC).

## Results

### Model selection

Two models including RF and LR can get the sensitivity of 100% in training, validation and two testing datasets, while other models including MLP, DT, SGD and KNN cannot. What’s more, all other evaluations including accuracy, specificity, PPV and AUC of RFC are all better in both models (Table 2). Overall, RFC is the optimal model for PKU screening.

**TABLE 2:** Results of multi-classification models of PKU. And, the bold values represent better results than other models.

	Models	Accuracy (%)	Sensitivity (%)	Specificity (%)	PPV (%)	AUC (%)
Training	RF	99.39	**100.00**	99.38	**57.39**	99.94
	MLP	99.88	100.00	99.88	87.42	99.96
	DT	99.59	100.00	99.59	67.00	99.97
	SGD	98.28	96.97	98.29	32.08	99.24
	LR	99.13	**100.00**	99.12	**48.71**	99.85
	KNN	100.00	100.00	100.00	100.00	100.00
Validation	RF	99.29	**100.00**	99.28	**58.48**	99.92
	MLP	99.71	89.86	99.81	82.67	99.87
	DT	99.46	98.55	99.47	65.39	99.14
	SGD	97.83	100.00	97.81	31.65	99.02
	LR	98.94	**100.00**	98.93	**48.59**	99.87
	KNN	99.58	86.96	99.71	75.00	99.14
GPMCH_2021	RF	99.44	**100.00**	99.43	**52.94**	99.91
	MLP	99.44	88.89	99.50	53.33	99.91
	DT	99.36	100.00	99.36	50.00	99.75
	SGD	98.52	100.00	98.51	30.00	99.40
	LR	98.94	100.00	98.93	37.50	99.95
	KNN	99.65	88.89	99.72	66.67	99.92
NWCH	RF	99.99	**100.00**	99.99	**24.62**	100.00
	MLP	99.64	100.00	99.64	1.12	99.98
	DT	99.99	93.75	99.99	33.30	96.87
	SGD	99.97	93.75	99.97	10.87	99.96
	LR	97.06	**100.00**	97.06	**0.14**	100.00
	KNN	99.94	100.00	99.94	6.38	100.00

### Training and evaluation of the model

**FIGURE 2:**
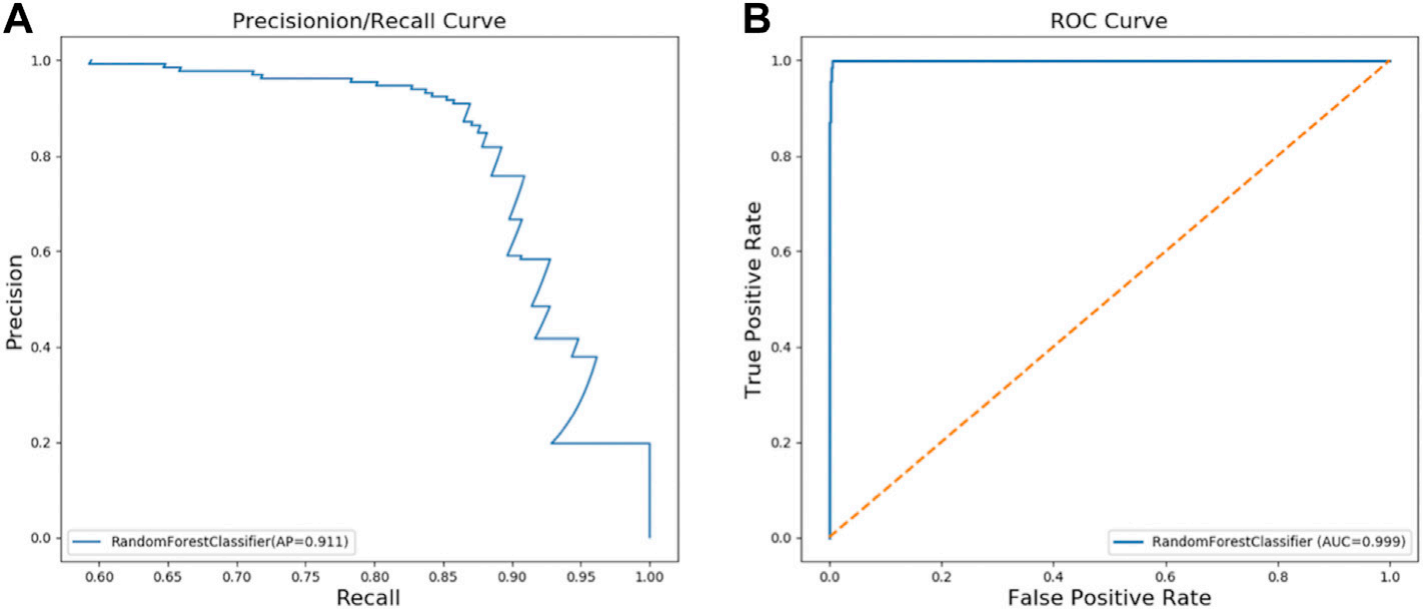
Two curves for PKU screening using RFC in the validation dataset: **(A)** PR curve; **(B)** ROC curve.

We constructed a RFC model to classify PKU patients and Non-PKU newborns. The final optimal RFC model used 72 trees in the forest, max depth 18, and min samples leaf 14. AP of the PR curve by RFC reaches 0.911 (Figure 2A), and AUC of the ROC curve reaches 0.999 (Figure 2B) in the validation dataset. These results show that the RFC is a reliable diagnostic tool for PKU screening.

**FIGURE 3:**
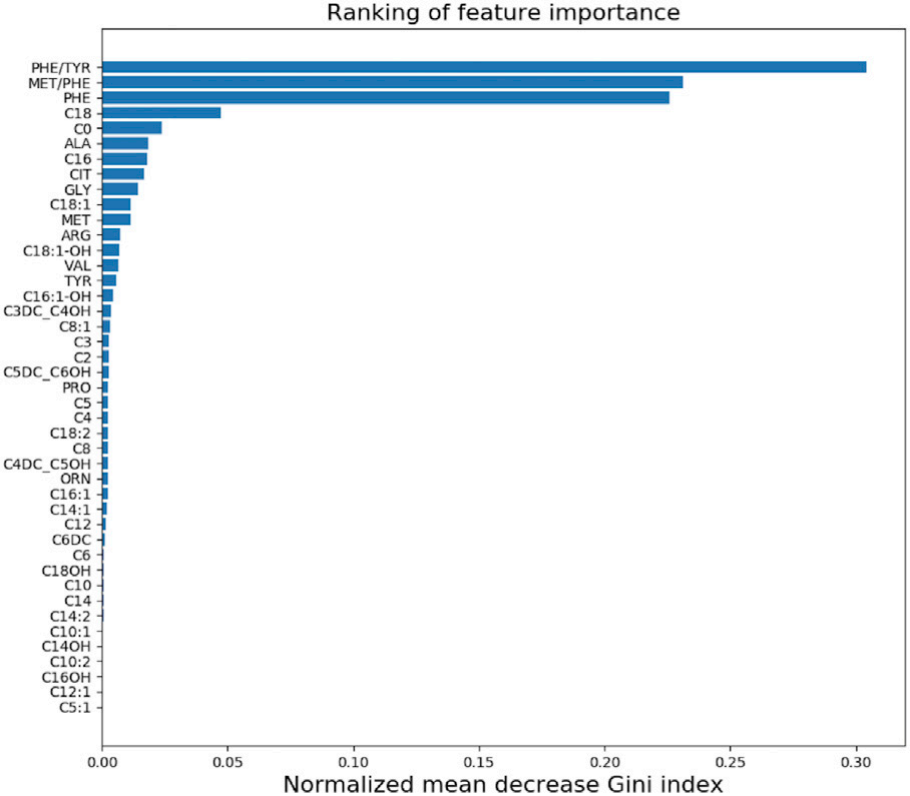
The ranking of 43 small molecule metabolites importance in our model.

Three of the top-ranked features including PHE/TYR, MET/PHE and PHE play the most important roles for RFC model. All the 43 features importance for the model construction of PKU screening can be seen in Figure 3.

### Validation of the model

In the validation dataset, PPV obtained for PKU screening by the traditional medical method (PHE>120 μ mol/L) is 17.7%. Using our model, PPV is significantly improved with a 3.3-fold increase to 58.48% (Pearson’s Chi-squared test, *p* < 2.2e-16). According to the traditional medical method for PKU screening, PPV of GPMCH_2021 dataset is 17.7% and that of NWCH dataset is 7.4%. PPV increase from 17.7% to 52.94% (Pearson’s Chi-squared test, *p* = 0.011) in the GPMCH_2021 dataset and from 7.4% to 24.62% (Pearson’s Chi-squared test, *p* = 0.0003) in the NWCH dataset (Table 3). It can be seen that RFC does significantly enhance the ability of PKU screening.

**TABLE 3:** Validation of our model.

Datasets	TP	FP	TN	FN	Sensitivity (%)	Specificity (%)	PPV (%)
Validation	69	49	6,743	0	100	99.28	58.48
GPMCH_2021	9	8	1,399	0	100	99.43	52.94
NWCH	16	49	392,144	0	100	99.99	24.62

### Comparison with the logistic regression model

In both of the testing datasets, we compared RFC with LRA3. RFC detected all patients, while LRA3 missed one PKU patient in the GPMCH_2021 (Table 4) and three in the NWCH dataset (Table 5). At the same time, Specificity and PPV also achieve good performance.

**TABLE 4:** Comparison with LRA3 in the GPMCH_2021 dataset.

GPMCH_2021	TP	FP	TN	FN	Sensitivity (%)	Specificity (%)	PPV (%)
Our model	9	8	1,399	0	100	99.43	52.94
LRA3	8	6	1,401	1	88.89	99.57	57.14

**TABLE 5:** Comparison with LRA3 in the NWCH dataset.

NWCH	TP	FP	TN	FN	Sensitivity (%)	Specificity (%)	PPV (%)
Our model	16	49	392,144	0	100	99.99	24.62
LRA3	13	28	392,165	3	81.25	99.99	31.71

## Discussion

Our model can both reduce the number of false positive cases and detect all the PKU patients during PKU screening. Sensitivity is 100% in two testing datasets, which means that none of PKU cases will be missed. In machine learning, there are many common classification models, such as MLP, DT, SGD, LR, KNN and RFC. Various indicators of the classification models are calculated, including accuracy, sensitivity, specificity, PPV and AUC. Comparing with these classification models, RFC showed clear advantages. In two testing datasets, PPV increased significantly compared with the traditional medical method. In the clinical setting, it is necessary to ensure that all PKU patients can be detected which means the sensitivity should be 100%. According to this rule, MLP and KNN methods show good results in the training dataset, but perform poorly in the validation and two testing datasets, where there is severe over-fitting. The DT method also shows excellent performance in the training dataset, but suffers from false negatives in the testing dataset and NWCH (Alexander, 2022). Some false negatives are also existed by LRA3, resulting in some PKU cases being predicted as negative. It is just an acceptable result in machine learning, but not to clinically acceptable.

In addition, Breiman (Breiman, 2001) pointed out that in the extremely imbalanced data, trees in random forest may contain few or none minority classes after bootstrapping, resulting in poor prediction performance for the minority classes. In our model, we set class weights for the extremely imbalanced data due to the large difference in the amount of data between positive and negative samples. In the tree induction procedure, class weights are used to weight the Gini impurity for finding the split (Chen et al., 2004), which is very important to the accuracy of the model.

Our study also has some shortcomings. Firstly, the number of positive samples in the testing dataset is not large enough for the very low incidence in southern China. For further development, it is necessary to increase negative and positive samples in the testing dataset to validate the model. Secondly, we found that the PPV of the NWCH dataset was lower than that of the GPMCH_2021 dataset, which may be related to the difference in the incidence rate between the north and the south. Since the incidence rate in the south is lower than that in the north and the penalty weight is calculated according to the proportion of positive and negative samples, the penalty weight of negative samples in the NWCH dataset is much greater than that of negative samples in the GPMCH_2021 dataset. We used the data of Gansu Province to train the model, there were more false positives and lower PPV when the NWCH dataset was the testing dataset. Finally, in low birth weight and premature newborns, the meaning of the measured value is often unclear, and there is no definite reference value so far, which is bound to have an impact on the prediction results.

In conclusion, machine learning-based random forest classifier can improve PKU screening performance with excellent sensitivity, FPR and PPV in two Chinese large populations. RFC is promising to be applied to neonatal PKU screening.

## Data Availability

The original contributions presented in the study are included in the article/Supplementary Materials, further inquiries can be directed to the corresponding authors.
