# Supplementary material for: Random forest classifier improving phenylketonuria screening performance in two Chinese populations
Source: Front Mol Biosci. 2022 Oct 11;9:986556. doi: 10.3389/fmolb.2022.986556 (PMC9592754; doi:10.3389/fmolb.2022.986556)
Supplement: Supplementary file 1 [file Table1.DOC]

Table S1. Descriptive statistics for 43 features in the total dataset.

| 43 Biomarkers | Mean | Std | Q25% | Q50% | Q75% | Min | Max |
| --- | --- | --- | --- | --- | --- | --- | --- |
| ALA | 297.71 | 94.47 | 237.78 | 281.98 | 338.06 | 51.25 | 4770.27 |
| ARG | 10.81 | 9.65 | 4.62 | 8.30 | 13.88 | 0.49 | 999.23 |
| CIT | 14.02 | 6.76 | 11.03 | 13.09 | 15.70 | 0.95 | 1208.53 |
| GLY | 439.86 | 121.19 | 360.34 | 425.21 | 501.99 | 35.36 | 3084.08 |
| MET | 20.72 | 7.49 | 16.46 | 19.73 | 23.74 | 1.85 | 1148.02 |
| ORN | 102.27 | 35.84 | 80.35 | 96.52 | 116.36 | 5.49 | 1390.62 |
| PHE | 52.94 | 43.78 | 44.08 | 50.08 | 57.36 | 3.34 | 3696.85 |
| PRO | 186.37 | 49.97 | 153.51 | 178.84 | 210.27 | 2.97 | 2042.17 |
| TYR | 104.83 | 43.55 | 76.32 | 96.46 | 123.16 | 0.57 | 1740.60 |
| VAL | 123.16 | 33.07 | 102.00 | 118.23 | 137.78 | 10.75 | 1172.23 |
| C0 | 23.05 | 9.52 | 17.15 | 21.47 | 27.01 | 1.86 | 1620.90 |
| C2 | 18.28 | 6.61 | 13.88 | 17.43 | 21.68 | 0.61 | 259.34 |
| C3 | 1.56 | 0.73 | 1.12 | 1.44 | 1.86 | 0.06 | 125.05 |
| C3DC_C4OH | 0.13 | 0.09 | 0.08 | 0.12 | 0.16 | 0.01 | 27.84 |
| C4 | 0.23 | 0.07 | 0.18 | 0.22 | 0.26 | 0.03 | 5.33 |
| C4DC_C5OH | 0.20 | 0.24 | 0.15 | 0.18 | 0.22 | 0.04 | 108.04 |
| C5 | 0.11 | 0.05 | 0.08 | 0.10 | 0.12 | 0.01 | 5.34 |
| C5:1 | 0.02 | 0.01 | 0.01 | 0.01 | 0.02 | 0.00 | 0.75 |
| C5DC_C6OH | 0.14 | 0.05 | 0.10 | 0.13 | 0.17 | 0.01 | 5.17 |
| C6 | 0.05 | 0.02 | 0.04 | 0.05 | 0.06 | 0.00 | 2.80 |
| C6DC | 0.14 | 0.06 | 0.10 | 0.14 | 0.17 | 0.01 | 10.49 |
| C8 | 0.06 | 0.04 | 0.05 | 0.06 | 0.08 | 0.00 | 17.57 |
| C8:1 | 0.15 | 0.07 | 0.11 | 0.14 | 0.18 | 0.01 | 3.29 |
| C10 | 0.08 | 0.04 | 0.06 | 0.08 | 0.10 | 0.00 | 1.97 |
| C10:1 | 0.09 | 0.04 | 0.06 | 0.08 | 0.11 | 0.00 | 2.33 |
| C10:2 | 0.02 | 0.03 | 0.01 | 0.02 | 0.02 | 0.00 | 19.28 |
| C12 | 0.09 | 0.05 | 0.06 | 0.08 | 1.11 | 0.00 | 2.06 |
| C12:1 | 0.06 | 0.04 | 0.04 | 0.06 | 0.08 | 0.00 | 1.48 |
| C14 | 0.17 | 0.06 | 0.13 | 0.16 | 0.20 | 0.00 | 3.67 |
| C14:1 | 0.09 | 0.05 | 0.06 | 0.08 | 0.10 | 0.01 | 13.21 |
| C14:2 | 0.02 | 0.01 | 0.02 | 0.02 | 0.03 | 0.00 | 3.70 |
| C14OH | 0.02 | 0.01 | 0.01 | 0.01 | 0.02 | 0.00 | 0.31 |
| C16 | 2.78 | 1.16 | 2.00 | 2.74 | 3.50 | 0.00 | 13.33 |
| C16:1 | 0.16 | 0.08 | 0.10 | 0.15 | 0.21 | 0.00 | 2.44 |
| C16:1-OH | 0.04 | 0.01 | 0.03 | 0.03 | 0.04 | 0.00 | 0.87 |
| C16OH | 0.02 | 0.01 | 0.02 | 0.02 | 0.03 | 0.00 | 0.69 |
| C18 | 0.81 | 0.28 | 0.62 | 0.78 | 0.97 | 0.01 | 4.18 |
| C18:1 | 1.34 | 0.41 | 1.07 | 1.31 | 1.58 | 0.04 | 7.84 |
| C18:1-OH | 0.02 | 0.01 | 0.02 | 0.02 | 0.03 | 0.00 | 2.49 |
| C18:2 | 0.23 | 0.12 | 0.15 | 0.21 | 0.29 | 0.01 | 4.11 |
| C18OH | 0.02 | 0.01 | 0.01 | 0.01 | 0.02 | 0.00 | 0.87 |
| MET/PHE | 0.41 | 0.13 | 0.33 | 0.39 | 0.47 | 0.01 | 13.95 |
| PHE/TYR | 0.58 | 2.16 | 0.41 | 0.52 | 0.66 | 0.05 | 1205.20 |
